# Impact of Archwire Presence and Adhesive Removal Techniques on Enamel Crack Formation During Orthodontic Bracket Debonding: An In Vitro Study

**DOI:** 10.1002/cre2.70486

**Published:** 2026-10-22

**Authors:** Samane Bakhshi, Reza Ghorbanipour, Ghazaleh Ahmadizenouz, Seyedali Seyedmajidi, Maysam Mirzaie

**Affiliations:** ^1^ Student Research Committee Babol University of Medical Sciences Babol I.R. Iran; ^2^ Dental Materials Research Center, Health Research Institute Babol University of Medical Sciences Babol I.R. Iran; ^3^ Oral Health Research Center, Health Research Institute Babol University of Medical Sciences Babol I.R. Iran

**Keywords:** dental enamel, dental instruments, dental polishing, orthodontic brackets

## Abstract

**Objectives:**

Enamel cracks are a common complication following orthodontic bracket debonding and the removal of adhesives. This study investigated whether the presence of an archwire during debonding and the use of turbine (high‐speed) versus angle (low‐speed) tungsten carbide burs affect the number, length, or orientation of enamel cracks.

**Material and Methods:**

In this in vitro experimental study, 64 extracted human premolars were randomly assigned to four groups (*n* = 16): with or without an archwire during debonding, and adhesive removal using either a high‐speed (turbine) or a low‐speed (angle) tungsten carbide bur. Stereomicroscopic images were taken before bonding and after adhesive removal to assess the number, length, and direction of enamel cracks. Adhesive Remnant Index (ARI) scores were also recorded. Data were analyzed using paired *t*‐tests, ANOVA, and Chi‐square tests (*α* = 0.05).

**Results:**

All groups exhibited a statistically significant increase in both the number and length of enamel cracks following bracket debonding and adhesive removal (*p* < 0.001). However, no significant differences were observed between the groups in terms of the increase in crack length (*p* = 0.166), crack number (*p* = 0.628), or ARI scores (*p* = 0.89).

**Conclusions:**

While orthodontic bracket debonding and adhesive removal significantly increase enamel cracking, the presence of an archwire and the type of tungsten carbide bur do not seem to affect the severity or pattern of crack formation. Therefore, clinical emphasis should be placed on careful debonding techniques and appropriate final polishing to minimize enamel damage.

## 1 Introduction

Enamel cracks are a clinically significant complication that may compromise tooth integrity, resulting in hypersensitivity, pain, and, in severe cases, structural failure. They may develop under continuous physiological loading or pathological factors, including trauma and iatrogenic dental procedures. Cracked tooth syndrome is defined as an incomplete fracture of indeterminate depth originating from the crown and potentially extending into the pulp and periodontal ligament. In orthodontic treatment, inappropriate bonding and debonding procedures may contribute to enamel damage, including surface scarring, residual adhesive, enamel loss, and the formation or propagation of enamel cracks (Torabinejad et al. 2020).

Debonding—the removal of brackets and bands at the end of fixed orthodontic treatment—can cause varying degrees of pain and enamel surface damage. One influential factor in the extent of damage is the presence or absence of the archwire within the bracket slot during debonding. The presence of the archwire may impede full plier function, necessitating greater force to break the bond, thereby increasing the risk of damage to the target tooth and adjacent teeth (Iqbal et al. 2023).

Following bracket removal, variable amounts of adhesive resin remain on the enamel surface, necessitating complete removal. Residual adhesive not only causes surface roughness that promotes plaque accumulation (Arbutina et al. 2020) but may also discolor over time, negatively impacting dental esthetics (Claudino et al. 2018). Studies have demonstrated that debonding, particularly when failure occurs at the enamel‐resin interface, predisposes to enamel cracking (Zachrisson et al. 2012).

Various methods for resin removal and enamel surface smoothing have been proposed, including tungsten carbide burs, polishing discs, rubber polishers, ultrasonic instruments, and newer technologies such as lasers and air abrasion (Dalaie et al. 2020; Sadri et al. 2021). However, due to the enamel's lower hardness compared to abrasive tools, a completely safe method for resin removal without enamel damage has yet to be established. The key challenge, therefore, is to remove adhesive resin thoroughly while minimizing damage and restoring the enamel to a near pre‐treatment condition (Cochrane et al. 2017).

Diagnosing cracked teeth can be challenging due to the variability of symptoms (Banerji et al. 2010). Common diagnostic methods include visual examination (Kim et al. 2020), periodontal probing and pulp vitality testing (Gopikrishna et al. 2009), staining techniques (Ghorbanzadeh et al. 2013), transillumination (Clark et al. 2003), and computed tomography (Landrigan et al. 2010). Early diagnosis is critical, as timely intervention can prevent crack propagation, microleakage, pulp involvement, periodontal complications, and cusp fractures (Kahler 2008).

Prevalence estimates in adults aged 30 to 50 range between 34% and 74%, with a higher incidence reported in females compared to males (Hasan et al. 2015; Hilton et al. 2018). Considering the high prevalence of enamel cracks in the adult population, in vitro studies are useful for clarifying the mechanisms that may initiate or aggravate these cracks during clinical procedures such as orthodontic bracket debonding.

Given the clinical importance of preventing enamel damage during bracket debonding and adhesive removal, this study aimed to investigate the effect of archwire presence during debonding and to compare different resin removal techniques on the extent and formation of enamel cracks. Although various methods have been suggested for adhesive removal, their comparative effects on enamel integrity—particularly regarding crack initiation and propagation—remain inconclusive. Furthermore, the influence of archwire retention during debonding, despite being a common clinical scenario, has not been sufficiently explored in terms of its mechanical consequences on enamel surfaces. This knowledge gap highlights the need for a systematic evaluation of both the mechanical context (with or without archwires) and post‐debonding polishing techniques to inform clinical protocols that minimize iatrogenic damage. The findings of this study may provide evidence‐based recommendations for orthodontic practitioners seeking to optimize enamel preservation during treatment completion, ultimately contributing to improved long‐term outcomes in dental health and esthetics.

## 2 Materials and Methods

### 2.1 Study Design and Sample Preparation

The inclusion criteria in this in vitro experimental study were de‐identified human premolars extracted for orthodontic purposes with intact labial enamel surfaces. Teeth with caries, restorations, or visible extraction‐related damage affecting the labial enamel surface were excluded. As the specimens could not be linked to individual donors, demographic information, including age and sex, was unavailable.

All teeth were stored immediately after extraction in a 0.5% chloramine‐T solution for 1 week to facilitate disinfection and were then kept in distilled water until the start of the experiment. For specimen disinfection and storage, the procedures described in ISO/TS 11405:2015 were followed. At the time of conducting the experimental procedures, ISO/TS 11405:2015 was the applicable version available for this purpose (ISO, P. 2015).

To ensure an unbiased distribution, the selected teeth were randomly allocated into the study groups using a simple randomization method by computer‐generated sequence. To ensure standardization across specimens, the same protocols for specimen preparation, bonding, bracket positioning, light curing, thermocycling, aging, debonding, adhesive removal, and crack assessment were applied to all experimental groups. The specimens were randomly allocated to the study groups, and the crack assessment was performed by an examiner blinded to group allocation.

### 2.2 Sample Size Calculation

The sample size was calculated based on the primary outcome (change in enamel crack length) using data reported by Heravi et al. (2008). Assuming a two‐sided significance level of 5% (*α* = 0.05), a statistical power of 80% (*β* = 0.20), and comparison of two independent groups, the required sample size was calculated using the following formula:

$$n=\frac{{\left(Z_{1-\alpha /2}+Z_{1-\beta }\right)}^{2}(S_{1}^{2}+S_{2}^{2})}{{\left(\mu_{1}-\mu_{2}\right)}^{2}}$$

Substituting the reported values:

$$\frac{{\left(1.96+0.84\right)}^{2}(3.1^{2}+3.2^{2})}{{\left(11.3-8.1\right)}^{2}}=15.2≅16$$

Accordingly, a minimum of 16 specimens per group was required.

### 2.3 Measurements

The buccal surface of each tooth was cleaned with a pumice‐water slurry using a disposable prophy brush (Disposable Prophy Brush, TPC, China) attached to a low‐speed handpiece. Surfaces were rinsed with water and air‐dried with oil‐free air for 10 s.

Enamel surfaces were examined under a stereomicroscope (Dewinter, Italy) at 10–20× magnification equipped with a digital camera. After precise focusing, images of the buccal surfaces were captured. The number, length, and orientation of enamel cracks relative to the tooth's long axis (vertical, horizontal, oblique, or mixed) were analyzed using Dewinter Capture Pro 4.6 software with 0.1 mm accuracy (Figure 1). Microscopic evaluations were performed by a laboratory operator who was blinded to the sample groups. Crack orientation was classified as follows:

- Vertical: 0°–30° relative to the tooth long axis
- Oblique: 31°–45°
- Horizontal: 46°–90°
- Mixed: cracks exhibiting direction changes along their length (Heravi et al. 2008)

**Figure 1:**
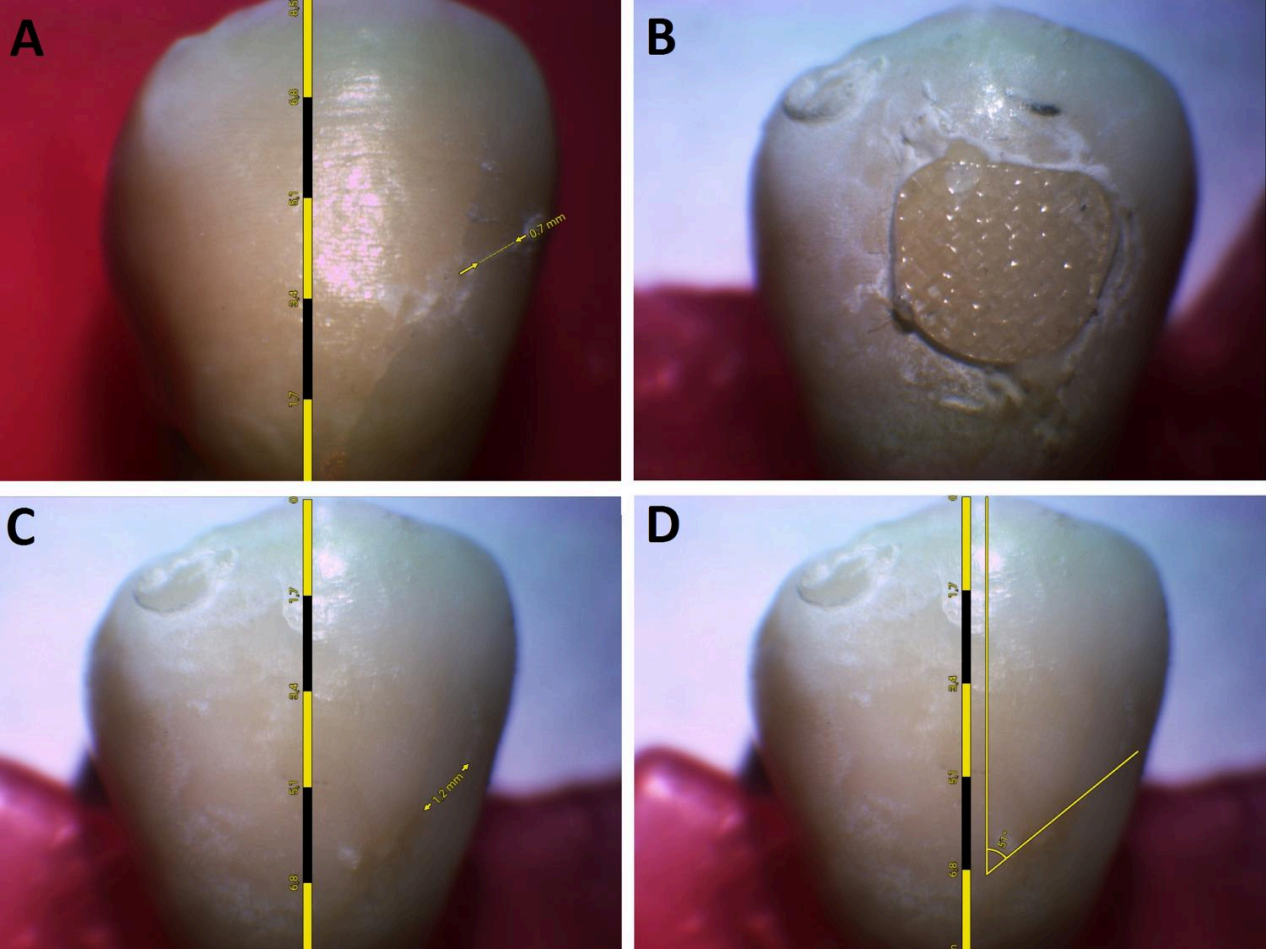
Stereomicroscopic evaluation of enamel surface cracks and adhesive remnants. Crack number, length (mm), and orientation (°) were assessed relative to the tooth long axis. (A) Baseline enamel surface before bracket bonding. (B) Enamel surface after debonding for adhesive remnant index (ARI) assessment. (C) Enamel surface after adhesive removal and polishing, showing linear crack measurements. (D) Corresponding angular assessment of crack orientation relative to the tooth's long axis.

### 2.4 Bracket Bonding

The enamel was etched for 30 s using 37% phosphoric acid gel (Morva‐Etch, Morvabon, Iran), rinsed for 20 s, and air‐dried for 20 s until a white, frosted surface appeared. A layer of Adper Single Bond 2 adhesive (3M ESPE, St. Paul, USA) was applied with a microbrush (Ultrafine, TPC, China), left for 20 s, and light‐cured with an LED unit (Woodpecker, China) at 1000 mW/cm^2^.

Edgewise metal premolar brackets (0.022‐inch slot, American Orthodontics, USA) were positioned approximately 4 mm from the cusp tip on the buccal surface. BracePaste adhesive (American Orthodontics, USA) was used for bonding, and each bracket was pressed with 300 g force using a force gauge (Dentaurum, Germany). Excess adhesive was removed with a probe tip. Light curing was performed for a total of 40 s (10 s from each of the mesial, distal, occlusal, and gingival directions) (Samadi et al. 2019). Although this curing time is longer than what is routinely applied in some clinical settings, it was intentionally chosen to ensure more uniform polymerization across all specimens and to reduce the influence of operator‐related factors or variations in light intensity. To simulate oral conditions, samples were incubated (LTE Scientific Ltd., OL37, Great Britain) in distilled water at 37°C for 24 h, followed by thermocycling (Nemo Fanavaran, Mashhad, Iran) for 1000 cycles between 5°C and 55°C water baths (30 s each, 10 s transfer time). According to ISO 11405 and previous literature on thermocycling, 500 cycles are typically regarded as representing short‐term aging of dental materials. Additionally, 10,000 cycles are suggested to roughly correspond to 1 year of clinical use. This implies that 1000 cycles fall within the range of early aging simulation (Gale and Darvell 1999; Ghavami‐Lahiji et al. 2018; ISO, P. 2015). For aging, the specimens were maintained in distilled water at 37°C for 1 month in an IP60 incubator (LTE Scientific Ltd., Great Britain), with a temperature accuracy of ±0.1°C. The incubator was set to 37°C throughout the aging period (Ryf et al. 2012).

### 2.5 Group Design

Sixty‐four samples were randomly divided into two groups of 32. In each group, teeth were mounted on 8 dental arches, and in half of the samples, a 0.018‐inch stainless steel archwire was engaged in the bracket slots. Each group was further randomly split into two subgroups of 16 for adhesive removal with either:

- High‐speed (NSK, Tokyo, Japan) 12‐fluted tungsten carbide bur (C379‐FG‐023, Komet, Germany)
- Low‐speed (NSK, Tokyo, Japan) 12‐fluted tungsten carbide bur (C379‐RA‐023, Komet, Germany)

Brackets were debonded using peeling force and a two‐blade bracket remover tool (Dentaurum, Germany) (Cochrane et al. 2017).

After debonding, the Adhesive Remnant Index (ARI) was recorded as:

- 0: No adhesive left on the tooth
- 1: Less than half the adhesive remains
- 2: More than half the adhesive remains
- 3: All adhesive remaining on the tooth surface (Heravi et al. 2008)

Before adhesive removal, the enamel surface was re‐imaged. Adhesive was then removed using the assigned bur until no residue was visible under unit light examination (dry and wet) and tactilely with a probe.

Finally, the buccal surfaces were re‐examined by a stereomicroscope, and enamel cracks were reassessed for number, length, and pattern (Figure 1).

### 2.6 Statistical Analysis

Statistical analyses were performed using SPSS version 26 (IBM Corp., Armonk, NY, USA). Data normality was assessed using the Shapiro–Wilk test. Continuous variables are presented as mean ± standard deviation. Within‐group comparisons of enamel crack length and crack number before and after debonding were performed using paired *t*‐tests. The changes (post‐debonding minus pre‐debonding values) were then compared between the study groups using one‐way analysis of variance (ANOVA). The distribution of Adhesive Remnant Index (ARI) scores among groups was analyzed using the Chi‐square test. Statistical significance was established at *p* < 0.05.

### 2.7 AI Usage Declaration

Perplexity AI (powered by Grok 4.1) was used to refine sentence structure in portions of the text during editing. No research data, analysis, or scientific content was generated by AI. All content was author‐verified.

## 3 Results

The mean total length of enamel cracks before bonding and after debonding and adhesive removal in each study group is summarized in Table 1. As shown, there was a statistically significant increase in crack length in all groups after the procedures (*p* < 0.001).

**Table 1:** Comparison of total length of enamel cracks (mm) before bracket bonding and after adhesive removal and polishing across the study groups.

Group	Time	*N*	Mean	Std. deviation	95% Confidence interval for mean		Min.	Max.	*p* value^a^
					Lower bound	Upper bound			
No wire/Angle	Before	16	7.59	2.68	6.17	9.02	0	12.1	< 0.001
	After	16	10.22	2.46	8.91	11.53	6	14.6	
No wire/Turbine	Before	16	7.73	3.67	5.77	9.68	0	12.2	< 0.001
	After	16	12.04	3.59	10.13	13.95	0	14.8	
Wire/Angle	Before	16	7.89	2.85	6.37	9.41	0	11.2	< 0.001
	After	16	12.19	2.54	10.84	13.55	7.3	15.3	
Wire/Turbine	Before	16	9.1	2.62	7.7	10.5	4.2	13	< 0.001
	After	16	13.44	2.43	12.14	14.73	9.9	16.8	

^a^ Paired samples *t*‐test.

The mean number of enamel cracks before bonding and after debonding in each group is presented in Table 2. A significant increase was observed in all groups (*p* < 0.001).

**Table 2:** Comparison of the mean number of enamel cracks before bracket bonding and after adhesive removal and polishing across the study groups.

Group	Time	*N*	Mean	Std. deviation	95% Confidence interval for mean		Min.	Max.	*p* value^a^
					Lower bound	Upper bound			
No wire/Angle	Before	16	2.38	1.31	1.68	3.07	0	5	< 0.001
	After	16	3.06	1.53	2.25	3.88	1	6	
No wire/Turbine	Before	16	2.25	1.13	1.59	2.91	0	4	< 0.001
	After	16	3.44	1.15	2.82	4.05	0	5	
Wire/Angle	Before	16	2.25	1.13	1.65	2.85	0	4	< 0.001
	After	16	3.25	1	2.72	3.78	1	5	
Wire/Turbine	Before	16	2.44	1.21	1.79	3.08	1	5	< 0.001
	After	16	3.56	1.31	2.86	4.26	2	6	

^a^ Paired samples *t*‐test.

Table 3 shows the frequency distribution of crack patterns (vertical, oblique, horizontal, and mixed) before bonding and after debonding across study groups, including the crack count for each pattern at both time points.

**Table 3:** Frequency distribution of enamel crack patterns and crack counts across study groups before bonding and after adhesive removal and polishing.

Crack pattern	Time	Crack count	Group			
			No wire/Angle	No wire/Turbine	Wire/Angle	Wire/Turbine
Vertical	Before	0	8	4	6	8
		1	8	11	10	7
		2	0	1	0	1
	After	0	7	3	4	5
		1	8	10	12	11
		2	1	3	0	0
Horizontal	Before	0	13	15	14	16
		1	3	1	2	0
	After	0	13	15	14	15
		1	3	1	2	1
Oblique	Before	0	10	9	11	12
		1	6	7	5	4
	After	0	10	7	10	8
		1	6	9	6	7
		2	0	0	0	1
Mixed	Before	0	3	4	4	0
		1	6	9	5	7
		2	6	3	7	8
		3	1	0	0	1
	After	0	1	1	0	0
		1	5	4	2	3
		2	6	8	12	7
		3	3	3	2	5
		4	1	0	0	1

The mean increase in total crack length following debonding and adhesive removal was not statistically different among the study groups (*p* = 0.166), as shown in Table 4.

**Table 4:** Comparison of the mean increase in total enamel crack length among groups following debonding and adhesive removal.

Group	*N*	Mean	Std. deviation	95% Confidence interval for mean		Min.	Max.	*p* value^a^
				Lower bound	Upper bound			
No wire/Angle	16	2.63	2.09	1.51	3.74	0	7.1	0.166
No wire/Turbine	16	4.31	2.92	2.76	5.87	0	10.6	
Wire/Angle	16	4.3	2.87	2.77	5.83	0	9.1	
Wire/Turbine	16	4.34	2.25	3.14	5.54	0.3	8.2	

^a^ ANOVA.

Similarly, no significant differences were observed among the groups regarding the mean increase in the number of enamel cracks after debonding (*p* = 0.628) (Table 5).

**Table 5:** Comparison of the increase in crack numbers among groups after debonding and adhesive removal.

Group	*N*	Mean	Std. deviation	95% Confidence interval for mean		Min.	Max.	*p* value^a^
				Lower bound	Upper bound			
No wire/Angle	16	2.25	0.93	1.75	2.75	0	3	0.628
No wire/Turbine	16	2.31	0.7	1.94	2.69	1	3	
Wire/Angle	16	2.5	0.73	2.11	2.89	1	3	
Wire/Turbine	16	2.13	0.89	1.65	2.6	0	3	

^a^ ANOVA.

Figure 2 illustrates the frequency of cases with newly developed enamel cracks following debonding and adhesive removal. No statistically significant difference was found among the study groups (*p* = 0.503).

**Figure 2:**
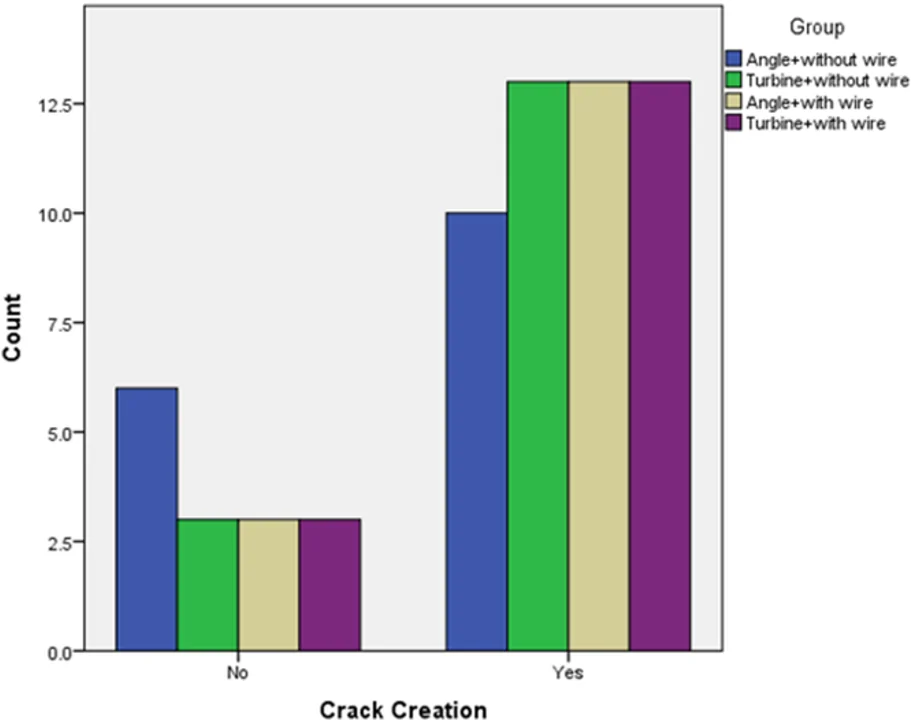
Comparison of the frequency of cases showing new enamel crack formation after debonding and adhesive removal across the study group.

The distribution of ARI scores across groups is presented in Table 6. No statistically significant difference was observed in ARI scores among the groups (*p* = 0.89).

**Table 6:** Frequency distribution of adhesive remnant index (ARI) scores after bracket debonding across the study groups.

Group		No wire/Angle	No wire/Turbine	Wire/Angle	Wire/Turbine	Total
ARI core						
0	Count	1	0	0	1	2
	% within ARI score	50%	0%	0%	50%	100%
	% within group	6.3%	0%	0%	6.3%	3.1%
1	Count	2	2	2	2	8
	% within ARI score	25%	25%	25%	25%	100%
	% within group	12.5%	12.5%	12.5%	12.5%	12.5%
2	Count	5	7	4	7	23
	% within ARI score	21.7%	30.4%	17.4%	30.4%	100%
	% within group	31.3%	43.8%	25%	43.8%	35.9%
3	Count	8	7	10	6	31
	% within ARI score	25.8%	22.6%	32.3%	19.4%	100%
	% within group	50%	43.8%	62.5%	37.5%	48.4%
*p* value*		0.89				64

* Chi‐square.

## 4 Discussion

This in vitro study investigated the effect of archwire presence during orthodontic bracket debonding and the type of adhesive removal tool (high‐speed turbine vs. low‐speed angle tungsten carbide burs) on the formation of enamel cracks. Results demonstrated a significant increase in both the number and length of enamel cracks after debonding and resin removal. However, no statistically significant differences were observed between groups regarding crack length and crack number or ARI scores, indicating that neither the presence of archwire nor the type of resin removal tool significantly influenced the extent of enamel damage. Although this study was conducted in vitro, the observed increase in enamel cracks, particularly mixed‐orientation patterns, reflects the clinical challenges often seen in practice. These results suggest that orthodontic debonding and adhesive removal procedures can be considered potential factors for enamel crack initiation, which may contribute to the dental sensitivity and structural issues reported in adult patients.

When an archwire remains in place, brackets are mechanically interconnected, and during debonding, part of the force applied to the target bracket is distributed through the wire to adjacent brackets. This load‐sharing effect may reduce the localized force on the enamel of the target tooth and potentially protect it from enamel fracture; however, it could simultaneously transfer force to neighboring teeth, placing them at risk. Although some laboratory studies have reported lower enamel crack frequency in archwire‐present groups under certain conditions, the overall effect appears highly dependent on wire type (SS, NiTi), wire gauge, bracket design, debonding technique, and instrumentation (Al‐Huwaizi and Mohammed 2007; Fan et al. 2017).

Previous studies have similarly emphasized that debonding and resin polishing inherently increase the likelihood of microcrack formation, especially when adhesive–enamel interface failure occurs. For instance, Paolone et al. (2023) reported enamel loss volumes up to 0.61 mm^3^ post‐debonding, which remains unavoidable even when using gentler instruments; nonetheless, carbide burs with multiple blades combined with multi‐step polishing systems result in minimal damage. Janiszewska‐Olszowska et al. (2016) found that tools such as one‐step polishers and adhesive removers produce smoother surfaces and less damage compared to traditional burs.

In this study, in addition to crack number and length, crack orientation was assessed. Vertical cracks were the most prevalent pattern observed both before and after debonding, while horizontal cracks were relatively rare and showed no significant frequency change. Conversely, mixed‐orientation cracks (those exhibiting directional changes along their path) increased in frequency after adhesive removal. This finding may reflect a more complex stress distribution and force concentration within the enamel structure following mechanical interventions.

Previous research supports that crack direction reflects the nature of applied forces; vertical and oblique cracks are typically associated with peel‐off and torque forces, whereas lateral or tensile forces tend to produce mixed or deflected cracks (Ahangar Atashi et al. 2018; Cochrane et al. 2017). The increased mixed cracks may also result from cumulative damage due to heat, aging, and residual stresses within the resin–enamel interface, which are difficult to detect with the naked eye. Since mixed cracks can propagate unpredictably, their increase post‐debonding and polishing may indicate a higher potential for long‐term microscopic failures, especially if final polishing is inadequate, as a greater number of microcracks may act as stress concentrators within the enamel structure. Clinical findings report that multi‐directional cracks under occlusal loading are associated with pain symptoms, including cold sensitivity and biting discomfort (Hilton et al. 2018).

A 2014 systematic review by Janiszewska‐Olszowska cautioned against the use of Arkansas stones, diamond discs, or lasers due to their higher risk of enamel damage, recommending multi‐step tungsten carbide burs instead (Grocholewicz 2014). Recent studies reinforce this, showing that tungsten carbide burs produce less surface roughness and more effective resin removal than zirconia burs or white stones (Gorassini et al. 2024; Thawaba et al. 2023).

Beyond instruments, other factors, such as bracket base design, may contribute to microscopic damage. Ahangar Atashi et al. demonstrated that brackets with Anchor Pylon bases induce longer cracks compared to mesh bases, although crack quantity did not differ significantly (Ahangar Atashi et al. 2018).

Regarding thermocycling as a simulation of the oral environment, Alsulaimani et al. (2024) reported no significant effect on ARI scores or new crack formation after thermocycling, although all groups in this study underwent thermocycling.

Advances in imaging techniques have greatly enhanced the understanding of enamel damage. For example, Hamdan et al. (2025) used Optical Coherence Tomography (OCT) to show that debonding increased crack length by approximately 20% and width by over 100%, with nearly half of the samples exhibiting new cracks—changes undetectable by conventional methods. Furthermore, recent 3D imaging with micro‐CT combined with deep learning algorithms has enabled precise identification of enamel crack patterns and pathways deep within tooth structure. Studies utilizing µCT scans and convolutional neural networks (CNN) successfully visualized complex crack networks at micron resolution in dentin and enamel (Dumbryte et al. 2023, 2022).

### 4.1 Limitations and Recommendations

Despite the rigorous control of laboratory conditions, including thermocycling, aging, and precise crack evaluation, this study has several limitations. First, the in vitro environment cannot fully replicate oral conditions, such as functional occlusal stresses, periodontal tissues, and biological factors. Second, only premolar teeth were included to ensure specimen standardization; since enamel thickness and structural characteristics vary among different tooth types (e.g., incisors or molars), these findings may not be directly generalizable to the entire dentition. Third, the specimen disinfection and storage procedures were based on ISO/TS 11405:2015, which has subsequently been withdrawn. Therefore, differences between this protocol and recommendations in the current version should be considered when interpreting the findings.

Methodologically, all microscopic evaluations were performed by a single operator; although blinding was strictly maintained to minimize operator bias, inter‐examiner reliability was not evaluated. Additionally, advanced imaging tools like OCT or micro‐CT that enable micrometer‐scale 3D crack detection were not extensively used here. Recent studies have demonstrated that OCT and micro‐CT are capable of revealing subsurface and microcracks that are not detectable under stereomicroscopic examination, thereby providing a more comprehensive evaluation of enamel damage (Dumbryte et al. 2023, 2022; Hamdan et al. 2025). Consequently, only surface crack length and number were quantified, while crack depth and volumetric micro‐structural variations remained unmeasured. Additionally, although disinfection in 0.5% chloramine‐T and storage in distilled water followed standard protocols, subtle alterations in enamel properties due to these agents cannot be entirely ruled out.

Furthermore, while the 1000 thermocycling cycles used in this study represent early aging simulation, future studies could employ more extensive thermocycling (e.g., 10,000 cycles) to simulate long‐term clinical aging. Moreover, further investigations are warranted to specifically elucidate how thermal fatigue and cycling duration affect enamel brittleness and its dynamic relationship with crack propagation.

Finally, future research should investigate newer resin removal methods, such as fiber‐reinforced burs, air abrasion, and laser techniques, using in vivo or clinical samples coupled with 3D imaging to allow detailed assessment of surface roughness, enamel structural damage, and long‐term effects, including dental sensitivity and plaque accumulation.

## 5 Conclusion

The findings of the present study revealed that although orthodontic bracket debonding and adhesive removal procedures significantly increased the number and length of enamel cracks, the presence of archwire during debonding and the type of tungsten carbide bur used (high‐speed vs. low‐speed) did not have a statistically significant effect on the extent of enamel damage. These results suggest that enamel cracking is primarily influenced by the debonding and mechanical resin removal processes themselves, rather than by factors such as archwire presence or the specific type of rotary instrument. Therefore, it is essential to employ safer debonding techniques, select minimally invasive tools, and implement appropriate surface polishing methods to preserve enamel integrity.

## Author Contributions

**Samane Bakhshi:** conducted the project work, collected data, and drafted the manuscript. **Reza Ghorbanipour:** supervised the project and revised the manuscript. **Ghazaleh Ahmadizenouz:** conceptualization, methodology, and visualization. **Seyedali Seyedmajidi:** formal data analysis and manuscript editing. **Maysam Mirzaie:** methodology and manuscript editing. All authors read and approved the final manuscript.

## Ethics Statement

This study was approved by the Ethics Committee of Babol University of Medical Sciences (IR.MUBABOL.HRI.REC.1403.121).

## Conflicts of Interest

The authors declare no conflicts of interest.

## Data Availability

The data are available upon reasonable request from the corresponding author.
